# Life after falls prevention exercise – experiences of older people taking part in a clinical trial: a phenomenological study

**DOI:** 10.1186/s12877-021-02037-9

**Published:** 2021-01-31

**Authors:** Susanne Finnegan, Julie Bruce, Kate Seers

**Affiliations:** 1 Warwick Clinical Trials Unit, University of Warwick, Gibbet Hill Road, Coventry, CV4 7AL UK; 2 Warwick Research in Nursing, Warwick Medical School, University of Warwick, Coventry, CV4 7AL UK

**Keywords:** Older people, Falls prevention, Exercise, Qualitative, Phenomenology

## Abstract

**Background:**

There is little evidence about the lived experience of older people who have completed a falls prevention exercise programme and their life beyond their intervention.

**Method:**

A phenomenological interview study with 23 participants (12 females), mean age 81 years (range 74–93 years), residing in their own homes across England, who had participated in a falls prevention exercise intervention within the Prevention of Falls Injury Trial (PreFIT). The aims were to explore their experiences of:

i. being in a clinical trial involving exercise.
ii. exercise once their falls prevention intervention had finished.

Interpretative data analysis was informed by van Manen’s (1997) framework for phenomenological data.

**Results:**

Analysis of interviews about experiences of participating in PreFIT and what happened once the falls intervention ended identified five themes: Happy to help; Exercise behaviours; “It keeps me going”; “It wasn’t a real fall”; and Loss. Participants did not continue their specific exercises after they had completed the intervention. They preferred walking as their main exercise, and none reported preventing falls as a motivator to continue exercising. Participant experiences suggest that they have their own ideas about what constitutes a fall and there is disparity between their interpretation and the definition used by healthcare professionals and researchers.

**Conclusion:**

Despite good intentions and perceived benefits, on-going participation in falls prevention exercises beyond a structured, supervised intervention was not a priority for these older people. Promoting continuation of falls prevention exercises post-intervention is just as challenging as promoting uptake to and adherence during exercise programmes.

**Supplementary Information:**

The online version contains supplementary material available at 10.1186/s12877-021-02037-9.

## Background

Falls are a common and serious health issue [1]. Gait, balance, and strength disorders have been identified as major risk factors for falling but are modifiable with appropriately designed exercise interventions, which can reduce both risk and rate of falling in community-dwelling older adults [2, 3].

As well as type of exercise intervention, evidence-based recommendations indicate that falls prevention exercise must be of adequate frequency, intensity, and duration [2]. The recommended exercise dose is 50 h over a six-month period, with long-term participation recommended to maintain accrued benefits [2].

Despite these recommendations, there is a shortage of evidence as to the long-term effects of exercise, maintenance of accrued benefits, or changes in physical activity, health behaviours and health status beyond the end of falls prevention exercise interventions [4, 5]. This gap in the evidence needs to be addressed to determine the long-term sustainability of exercise programmes and how best to promote exercise that can reduce rate and risk of falls, as a life-long intervention [6, 7].

There is also a lack of evidence examining falls and falls prevention interventions from the older person’s perspective [8, 9]. Qualitative studies are crucial to understand the ‘how and ‘why’ behind the (in)effectiveness of interventions [10]. Therefore, as part of a larger mixed methods research project entitled: Life After Falls prevention Therapy involving ExeRcise (LAFTER) [11], a phenomenological interview study was carried out to answer the following research question: What are the experiences of older people participating in the exercise intervention of the Prevention of Falls Injury Trial (PreFIT)?

The aims were to explore the experiences of:

i. being in a clinical trial involving exercise.
ii. exercise once their falls prevention intervention had finished.

## Methods

Residing within a naturalistic paradigm, this interview study was underpinned by the philosophy of Heidegger’s interpretive phenomenology [12]. The aim of which is to uncover the meaning of ‘Being’, by closely examining individuals’ lived experiences and the way in which things present themselves in order to obtain a greater understanding of everyday experiences [12]. Some of the key concepts of Heidegger’s phenomenology included in this interview study are described in more detail in Table 1.

**Table 1:** Heideggerian Concepts

Heideggerian Concept	Brief description
**Being-in-the-world**	An a priori state whereby a person and the world are inseparable. This co-constitution between humans and the entities that they encounter shapes the world they inhabit, whilst they simultaneously construct their world and existence from their own experiences and background [13, 14].
**Being with**	“Being-with” is a critical structure of a person’s Being-in-the-world and is what Heidegger refers to as solicitude. In everyday life, a person’s existence is one of “Being with” others [12].
**Authenticity**	In being with others, one assumes either an ‘inauthentic’ existence i.e. conformist or passive, defining oneself as everyone else might or an ‘authentic’ existence one chooses to live life with explicit awareness of their own Being and possibilities [14].
**Leaping in**	When a person ‘leaps-in’ for another, they “take over for the Other that which with (the other) is to concern himself” [12] (p.158) and may take control, potentially leading to dependency.
**Possibilities and Projection**	Heidegger claims that people should be understood in terms of possibility. This is not necessarily some future event but rather that a person *is* his or her possibilities and can be involved in or project themselves into current activities ‘for-the-sake-of’ one or several on-going ways to be [15].
**Useful things**	Useful things are entities that manifest in our everyday activities. They are determined by a person’s opinions and ideas about their own existence and show themselves in ways that are relevant and appropriate to those activities [15].
**Ready to hand**	Useful things are considered to be ‘ready-to-hand’ when they are used with such familiarity so as to almost withdraw from the activity i.e. a person who is engaged in a task does not actively think about what they are using or what they are doing, and the useful thing might be considered as an extension of the body and, therefore, remains unnoticed [14].
**Signs**	The world, like useful things which are often taken for granted or scarcely noticed, can become conspicuous via signs. Signs can be interpreted in a variety of ways but make an individual’s world more explicit to themselves and to others [15].

### Study sample

Participants eligible for the LAFTER study [11] were selected from two arms of the PreFIT study: advice only (*n* = 2575) and advice plus exercise (*n* = 2316). These participants were sent a follow-up postal survey at four years (range three to six years) after randomisation into the main trial. Survey respondents were asked to indicate their interest in taking part in an interview study. This provided the sampling frame to identify a purposive sample of up to 30 participants who had been randomised to the exercise intervention of PreFIT [11]. The aim was to incorporate a diverse range of experiences by sex, age, history of falling and by adherence to the falls prevention exercise intervention. Participants with severe cognitive impairment based on the clock drawing test in the survey questionnaire were excluded [11].

Interested participants were mailed an information sheet about the interview study. Upon receipt of signed consent, each participant was contacted by telephone to arrange a mutually agreeable time and place for their interview. At the time of interview, consent was reviewed to ensure/confirm capacity and willingness to participate [11].

### Data collection

In order to obtain individual, detailed accounts of the lived experience and the context shaping the experience for the participant, interviews were the most appropriate method of data collection [16]. Semi-structured interviews (Additional file 1) were undertaken to give participants direction and ensure the emerging findings were relevant to the research question [17]. All interviews were conducted by the lead researcher (SF) and were carried out in the participants’ homes. With consent, interviews were audio digitally recorded on an encrypted recorder, anonymised using pseudonyms and transcribed verbatim [11].

### Data analysis

Interpretative data analysis was conducted concurrently with data collection and informed by van Manen’s iterative and inductive framework for phenomenological data [17]. The framework involves the use of non-linear steps, including reading and re-reading transcripts, adding preliminary comments, highlighting noteworthy sections, and identifying and organising themes. Interpretation of the data was supported with notes and reflections organised in a research diary. These notes helped identify key concepts and themes that were common or disparate and warranted further investigation. The findings were discussed with the other authors (KS and JB) to review the emerging themes. The sample size was deemed sufficient when key themes had been revealed and no new concepts were emerging from the data [18] .

### Rigour

To ensure trustworthiness, balanced integration, openness, concreteness, resonance, and actualisation [19] were implemented. This involved making explicit what Heideggerian phenomenology is and how it fits with the research; disclosing the researcher’s forestructures (preconceptions); keeping a research diary to make notes immediately after each interview and using it to reflect on interview processes and interview transcripts.

## Findings

### Participant characteristics

In total, 23 participants were interviewed between three and six years after randomisation to PreFIT. Twelve (52%) participants were female and mean age at the time of interview was 81 years (range 74–93 years). Neary all (20 participants) were considered to be at higher risk of falling based on previous falls and history of balance difficulties. Interview duration ranged from 17 to 63 min.

### Themes

**Fig. 1:**
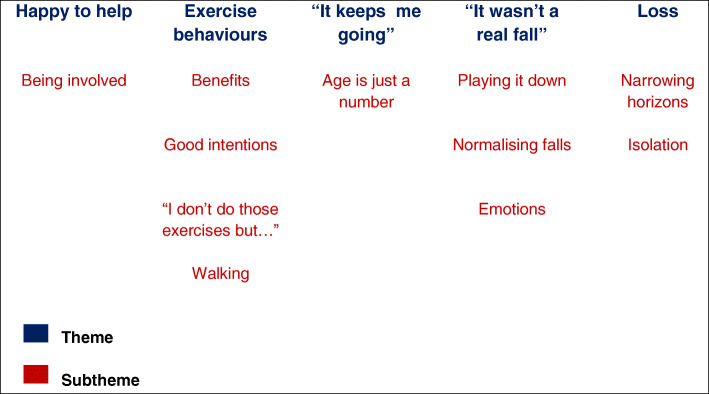
Schema of themes and subthemes

Interpretative analysis revealed five themes incorporating between one and four sub-themes (Fig. 1). The participants’ narratives included their reasons for participating and their involvement in the trial, but also conveyed in-depth experiences about falling, being active, life as an older person and the importance of remaining independent.

#### Theme 1: happy to help

Individuals explained their willingness to participate in the trial came about because they hoped that taking part in a research study would benefit others. They also decided to take part because the invitation came from their General Practitioner (GP):

“I’ve always gone along with everything that the [GP] surgery says…if they ask me to do something… yes I’ll do it…no other reason other than to say well ok it must be of some value to someone…you know…if it helps somebody” (Bob)*.*

Few participants mentioned falling as a reason for taking part in the study but those that did were still altruistic in their motivation, whilst hoping participation might also provide some personal benefit:

*“I just thought it can help anybody or even me then why not” (Ted).*

##### Being involved

The participants’ stories progressed from why they got involved to what being in the trial involved. This included completing questionnaires and falls diaries, which they described as straightforward and simple and the six-month exercise intervention which some declined to attend, partially completed, or completed in its entirety:

“I think initially it was answering all the questions… the questionnaire and then from that I was asked to participate in… a course of exercises…” (Deirdre)*.*

“A lady who told me she was an occupational therapist was going to… was going to be my carer or minder if you like to do a series of exercises regularly and gave me a book of exercises… to do at the time…” (Charles)*.*

#### Theme 2: exercise behaviours

This theme encapsulates not only the participants’ experiences of taking part in the PreFIT exercise intervention, but also other activities or exercise, their reasons for doing so and what exercise meant to them.

##### Benefits

Overall, the participants who completed the intervention reported it to be beneficial. Some described a general benefit whilst others described specific improvements in their joints, lower limb strength and balance. However, no-one mentioned the effect of exercise on preventing falls or reduction in falls:

“…I’ve felt the benefit from here on down… from my hips down…more than anything…and yes I enjoyed it at the end of it…” (Geoff)*.*

“I couldn’t see at the time exactly what it was going to do for me but now I can see the benefit of it… you know I just thought well I’ll do it… one of the other lady’s did comment on my balance being so good…well I said if it is then it’s thanks to this course I had to do for Warwick University” (Deirdre)*.*

Conversely, some participants reported little benefit, simply doing the exercises because they had been asked to:

“…I don’t think it made me feel any better or any worse I just did it” (Ann)*.*

“… it was a chore really… it wasn’t a pleasure… it was just something that I’ve got to get through” (Jane)*.*

For those experiencing benefits, the exercises might be considered what Heidegger calls ‘useful things’ that manifest in our everyday activities [15]. Whilst for others, the exercises manifested themselves differently and their experience was that they were only doing them because they were told to.

##### Good intentions

The participants described having good intentions about continuing with their exercises beyond the intervention. Several were still doing some of their exercises intermittently, but none were continuing with the complete prescribed programme (three times per week):

“I suppose I thought there was no need [to continue exercising] then I fell over!” (John)*.*

“I was only… only doing them because I was expected to do them, but I’ve dropped back in to my old [habits]…” (Molly)*.*

The participants’ were ‘authentic’ about their reasons for discontinuing with their exercise i.e. once their connection with the trial ended so did their motivation to continue with their prescribed exercises.

Some participants described other barriers to on-going exercise:

“…the more you do it she [therapist] said… I thought why don’t they listen to you… I am not going to get better I’m going to get worse you know” (Sarah)*.*

“I stopped doing them because my GP… he wasn’t at all happy about my doing this strength and balance… and he said, ‘no it’s too much for you…” (Charles)*.*

These quotations align with Heidegger’s concept of ‘being-with’ others who are ‘leaping in’ [12]. The healthcare professionals in these examples both dominated, taking over the experience, assuming they knew best for the participant.

##### “I don’t do those exercises but…”

Some participants felt they were already active enough. This subtheme highlights that older adults are active in many ways, choosing to exercise or be active in ways which are important, meaningful, and enjoyable to them:

“I don’t do those exercises, but I do… do exercises for…my knees but all in Pilates or Yoga…” (Kath)*.*

“…they have a gymnasium class in the morning and in the afternoon and if we stay in there’s golf on the carpet…and play bowls” (Tom)*.*

Participants were aware of the potential harm of inactivity, but highlighting personal choice, some described satisfaction in being sedentary:

“…well they always tell us the doctors and everybody that the worst thing you can do is just to sit in a chair and quite honestly I can’t think of anything nicer than just sitting in a chair…” (Sylvia)*.*

“I'm a person who quite enjoys sitting down (laughs)…what I don’t want to join in I don’t, you know” (Phyllis)*.*

##### Walking

The most common activity undertaken was walking and for some participants, the meaning of exercise or physical activity and walking were inextricably linked.

“It’s mainly walking for me… I don’t go to the gym or anything (laughs)” (Kath)*.*

“…well anytime you walk that’s physical activity isn’t it… I think most elderly people can’t do anything but walk really” (Jane)*.*

Walking is accessible, functional and can be incorporated into daily life. Therefore, walking is what Heidegger calls ‘ready-to-hand’ [15], i.e. forms part of their routine that does not need thinking about, and becomes inconspicuous as an exercise. Walking is considered as something necessary to remain an independent older adult and not be a burden to anyone else.

#### Theme 3: “It keeps me going”

Some participants described motivators for exercise and physical activity, with the most common being to “keep going”. They appreciate the need to exercise in order to maintain physical and mental health for the sake of remaining independent or doing the things they enjoy:

“…my idea of exercise is just continuing for as long as I can and that probably is my biggest fear that eventually I won’t be able to continue…” (Bob)*.*

“I want to keep as active as I can for as long as I can. Just keeping myself going and active… (Barry)*.*

##### Age is just a number

Many of the participants did not perceive their age as an obstacle to be able to “keep going”:

“…I don’t feel the age I am…I feel sorry for people I see who are the same age as me who are creeping about like geriatrics” (Kath)*.*

“…thinking you are old… you are old! I don’t feel… I don’t feel my age…and… hope I never will I am just grateful for what I’ve got” (Barbara)*.*

A lot of participants compared themselves to other older people, using others either as role models or conversely, as examples of not wanting to be like them. Heidegger suggests that a person’s way of Being is always an on-going concern and, therefore, should be understood in terms of ‘possibility’ [15]. By comparing themselves to others, these participants were ‘projecting’ themselves into possibilities:

“Well they won’t exercise or don’t want to exercise you know and they don’t move around enough…I don’t want it to be like that…” (Barry)*.*

“We’ve got some very good friends; he was 90 at the beginning of March and she’s nearly 90… incredible… they are our role models…they are going to Mexico this year… I think they are incredible” (Barbara)*.*

As the oldest participant, Molly described her experience of other people’s perception of her:

“I think that once people know your age…that they tend to want to do things for you… It makes me feel cross sometimes … I don’t like it…” (Molly)*.*

Similarly, Phyllis’ perceptions of age put her off using a wheeled frame even though she acknowledged that it may be of significant benefit to her mobility:

“My son was trying to say to me to get one of these pushers you know… I’m too proud to do that I don’t want to do that… that’s old ladies and I don’t want to be an old lady!” (Phyllis)*.*

A person’s world is often taken for granted but becomes conspicuous via ‘signs’ [12]. Signs associated with ageing e.g. physical appearance, walking aids etc. can be interpreted in different ways, but make the participants’ worlds more explicit. Once people knew their age or even just assumed their age based on the signs, they were often treated differently.

#### Theme 4: “It wasn’t a real fall”

Several participants said that their falls were not ‘real’. Often this was because they could describe a valid reason for their falls; these falls were their own fault and could have happened to anyone or their fall did not match the standardised definition of an “unexpected event in which the participants come to rest on the ground, floor, or lower level” (p.1619) [20] used by healthcare professionals and researchers:

“I’ve had no falls; I remember one day I didn’t class as a fall… I went down from one step onto the other and I went down… but I don’t think it was a fall as probably you would think” (Terry)*.*

Terry’s interpretation of his fall suggests that older people have their own ideas about what constitutes a fall. Similarly, Ann reported that she had never fallen but went on to explain “*I’ve never had a bad fall in my life” (Ann)*, equating a ‘bad fall’ with a ‘real fall’.

Often falls were described as a trip, slip or an accident occurring as the result of an external influence or environmental factor, and thus, not a ‘real’ fall:

“I have to be perfectly honest twice since I said I’d see you I’ve fallen over but not because I’ve fallen because I’ve slipped” (Kath)*.*

“I was hurrying one day… I fell and I realised that I was rushing forward, but it was really the soles on my shoes that had…just didn’t slide on the carpet” (Molly)*.*

Healthcare professionals and researchers would interpret these as ‘real’ falls and would initiate referral to intervention or treatment pathways regardless of the contributing factor i.e. problems with strength or balance requiring referral to physiotherapy or occupational therapy for home assessment. This contrasts with the perception of participants, where they blamed external factors as the reason for falling.

In line with Anne’s interpretation, participants describing a ‘real’ fall, did often describe it as a “bad fall”. These bad falls usually happened for no apparent reason, or involved difficulties with getting up or resulted in serious injury:

“I assume a fall is if you just fall over for no reason…” (Terry)*.*

“I don’t know what happened I just found myself on the ground… and I broke my arm very badly… that was a bad fall” (Phyllis)*.*

“…fell over the little kerb that was there and fell… down upside down… now that is the worst fall I’ve had” (Mary)*.*

##### Playing it down

Even when participants described ‘real’ or bad falls, some went on to justify the reasons for the fall. There were some contradictions between the description of the bad fall and the importance placed on it; suggesting that participants were playing it down:

“…definitely in the dark I wouldn’t have done it otherwise I mean gosh no I am not that stupid!” (Mary)*.*

“Actually, I broke my hip about five or six years ago… I fell over in the garden. I didn’t over balance or anything no … I was in the hospital nine days I think” (Pat)*.*

In contrast, those describing ‘real’ falls were less likely to play it down, describing lasting consequences:

“It just sort of got worse and I kept falling you see and…I did that [points to arm] ended up in [city] for a week having a skin graft” (Sarah)*.*

“… just slows me down terribly… I just can’t do things… I am aware that I am liable to fall and so there are things that I can’t do anymore (Sylvia)*.*

A ‘real’ fall can interrupt the flow of time, slowing it down or disrupting ways of Being previously taken for granted.

##### Normalising falls

For some participants, falls had become a normal part of life and were talked about as an acceptable or inevitable part of their everyday existence:

“… well you know I did have quite a lot of… well not a lot of falls but you know I noticed I was falling, and I was known for it… I had several falls in that month…” (Sylvia)*.*

“… about three times a week” (Tom)*.*

##### Emotions

Whether falls were seen as ‘real’ or not, participants often described how falling made them feel. Commonly, participants described emotions of anger, feeling foolish, surprise and upset. These emotions were in contrast to the somewhat relaxed attitudes exhibited when ‘playing it down’ or ‘normalising’ falls:

“…cross! (Laughs) I think how stupid can you get (laughs)” (Pat)*.*

“It shook me up a lot more than I thought it would to be honest… it disturbed me that it had happened… I suppose it has shown me that the older you get the more vulnerable you are” (John)*.*

This vulnerability is also apparent in another emotion that older people sometimes described, fear of falling. Several participants described ongoing wariness, caution and subsequent lack of confidence following their fall(s):

“It’s not that I’m frightened… I’m very wary about falling” (Donald)*.*

“It knocked your confidence to a degree; well to a big degree to be honest. I am much more conscious of walking now than I have ever been…” (John)*.*

A fall can result in a disruption of seeing the body as what Heidegger calls a ‘useful thing’ and might affect an older person physically and/or psychologically. Consequently, the body becomes conspicuous and less ‘ready-to-hand’ and, therefore, older people may start to worry about moving, walking, and falling, which in turn can lead to reduced mobility, activity restriction and social isolation.

#### Theme 5: loss

Some participants went on to describe other experiences of physical loss and functional limitations due to reduced mobility, ill health and falling.

##### Narrowing horizons

Restrictions in activity were conveyed by the participants with a sense of loss or giving up of things they used to do, places they used to go and people they used to see. This resulted in a narrowing of their horizons or what might be considered as a restriction in the projection of their possibilities:

“…going places…is quite daunting… one’s horizons are forever shrinking… it has restricted me from doing things… horizons just closing down… (Sylvia)*.*

Whereas some participants had become accustomed to or accepted a reduction in activity, others were not prepared to give in to limitations:

“I would… I should deteriorate fast… I would if I couldn’t get out… I’ve gotta get out off around the town” (Ted)*.*

“Completely shattered… I should be… I should be desperately unhappy… if I couldn’t get out” (Pat)*.*

##### Isolation

Although not directly linked to falls prevention and exercise, the loss of a spouse, relative or friend was described as a cause of significant change in activity and subsequently an increased risk of isolation, loneliness and potentially depression. Some participants were able to find ways to overcome this loss and used it as a motivator to be active, whereas others struggled:

“I don’t like being in the house on me own too long… I don’t like being in here on me own now…” (Ted)*.*

“When you are on your own you do get lonely and you have to do something… you know I make myself get out and do something if I’m feeling like that…” (Mary)*.*

##### Summary

The older people in this study were interviewed within the context of a falls prevention clinical trial involving exercise. All participants hoped that taking part in a research study would benefit others. They described some positive and negative experiences of engaging with falls prevention research but would recommend others to participate if invited.

Although most of the participants had fallen at some point, their experiences of falling fluctuated between an inconvenience which they described as of no real importance or consequence, to a life changing event. The participants acknowledged exercise and physical activity as being important but despite having completed strength and balance exercises which some found to be beneficial, they chose to undertake exercise or activities that they found more enjoyable and perceived to have more personal relevance.

## Discussion

This study interviewed a sample of older people who participated in a structured strength and balance falls prevention clinical trial completed in 2016 to ascertain experiences of participating in that research. Interviews explored the experiences of taking part in this research study and revealed issues that are important to people as they age.

Despite their good intentions and the perceived benefits of their falls prevention exercises, these participants stopped their specific exercises once their supervised programme ended. The reasons given for discontinuing are broadly similar to the problems commonly cited for uptake and adherence to group-based falls prevention exercise programmes [8, 21]. We also found similarities with the motivators and deterrents identified in a systematic review exploring the barriers and facilitators to continued participation in falls prevention exercises [5]. These include health issues; lack of time; lack of on-going supervision; no longer perceiving a need for exercise; no perceived benefit; and expressing a preference for other types of exercise or activity.

It has been suggested that there may be some disparity between the strategies that older people are willing to consider for preventing falls and those that are evidence-based i.e. older people are not motivated to undertake regular strength and balance exercises solely to help prevent falls [9, 22]. This was evident in our interview study, where no-one reported preventing falls as the primary motivator to exercise. Instead, reasons to exercise included improvements and maintenance of health and mobility linked to independence, enjoyment, and socialising. Therefore, our study participants did not consider specific falls prevention exercises to be relevant to their Being-in-the-world. These older adults exhibited individual agency or control; choosing what exercise or activities they felt were most appropriate for them, whether or not this concurred with their health professional’s advice.

Most commonly, and consistent with previous research [21, 23], study participants described a preference for walking as their main activity to keep going. This was because walking was perceived as ‘ready to hand’ [15] i.e. an accessible, convenient, and functional part of daily life. There is evidence that walking has significant health benefits, including improved cardiopulmonary fitness [24], but there is no evidence that walking alone is effective at reducing rate or risk of falls. Indeed, some research suggests walking has been associated with an increased risk of falls [3, 25].

The majority of our study participants had fallen previously, either during or since their involvement in the main PreFIT study [3]. Yet many of those interviewed did not consider these to be ‘real’ falls. Instead, these falls were often attributed to an immediate and definable cause such as a slip or trip, temporary inattention, or a totally unavoidable event, rather than as a result of individual risk or personal vulnerability [11, 26, 27]. Often slips and trips were not considered as ‘real’ falls but as a result of something that was out of their own control.

Although a commonly used definition of falls, developed from a multidisciplinary consensus conference involving user groups, worded as an “unexpected event in which the participants come to rest on the ground, floor, or lower level” (p.1619) [20] was used in the PreFIT study, data from our interview study suggest that this definition does not reflect older people’s way of Being-in-the-world. The definition is more clinically driven rather than having evolved from patients own perceptions and experiences [11]. This disparity in interpretation of the meaning of a fall has potential implications for how older people record or report falls. When exploring an older person’s falls history, clear and specific questioning is imperative, being mindful of careful exploration of any mention of slips, trips, or near misses, allowing time for older people to describe their experiences. We found evidence that falls are likely to be under-reported by older people.

The experience of falling varied from being a minor inconvenience of no real consequence, to a life changing event. Playing it down or normalising falls was common amongst our study participants and falls prevention only became a priority once a fall interfered with their everyday life or caused significant injury. Only then did it become a ‘real’ fall to the person.

Heidegger suggests that in everyday activity, people take a stand on the kind of thing they are Being, not necessarily by declaring to be one thing rather than another, but in engaging in some activities or tasks rather than others [28]. Therefore, these older people are taking a stand on the kind of thing they are Being by choosing to participate in the activities and tasks that they enjoy, or feel will be beneficial to their own personal goals. This usually includes remaining independent but was not related to preventing or reducing falls, regardless of whether perceived as ‘real’ or not.

### Strengths and limitations

This study based on Heideggerian philosophy explored the meaning of Being for older adults. Although qualitative research findings can be transferable, it is acknowledged that the study of lived experience is from the perspective of the individual experiencing the phenomenon [29, 30]. However, by describing the sample and the context that surrounds the data collection [11], this enables the reader to make judgements about transferability.

The strengths of our study include the large sampling frame for participant selection, sufficient sample size to ensure key themes were revealed and representation of older people with a history of falling. Our oldest participant was 93 years.

It is possible that others may generate different interpretations and themes from the data, but we have been rigorous and explicit in the methods used [19]. Given the lack of previous qualitative research into older adults’ experiences of life after completing a falls prevention exercise programme, a Heideggerian approach has allowed for a meaningful exploration of the phenomenon, providing a broad and rich understanding of participants’ experiences.

## Conclusion

Despite good intentions and knowledge of the perceived benefits from of exercise, on-going participation in falls prevention exercises beyond a structured and supervised intervention was not a priority for these older participants. They have their own motivations to exercise, choosing to participate in the activities that they enjoy, or think will be beneficial to their own personal goals. These goals largely include remaining independent for as long as possible as they age, but not preventing or reducing falls. Therefore, promoting long-term behavioural change by encouraging continuation of falls prevention exercises post-intervention has similar barriers to those highlighted when promoting uptake to and adherence to exercise programmes.

It is important that all the relevant stakeholders understand the importance of an individual older person’s agency and work with them to understand their needs and wants with regards to the role of exercise in maintaining health and independence and reducing the risk and rate of falls. Importantly, an older adult’s perception of what constitutes a ‘real’ fall differs from the professional definition and interpretation of falls by healthcare professionals. Thus, based on this study, for many older people, the trigger for participation in a falls prevention exercise programme may only be when older adults experience what they perceive to be a ‘real’ fall, interpreted as an event that may impact on their future independence.

## Supplementary Information

**Additional file 1.**

## Data Availability

The datasets used and/or analysed during the current study are available from the corresponding author on reasonable request.

## Abbreviations

GP: General Practitioner
LAFTER: Life After Falls prevention Therapy involving ExeRcise
PreFIT: Prevention of Fall Injury Trial
