# Supplementary material for: Life after falls prevention exercise – experiences of older people taking part in a clinical trial: a phenomenological study
Source: BMC Geriatr. 2021 Jan 31;21:91. doi: 10.1186/s12877-021-02037-9 (PMC7849142; doi:10.1186/s12877-021-02037-9)
Supplement: Supplementary file 1 — Additional file 1. [file 12877_2021_2037_MOESM1_ESM.docx]

**Additional file 1**

**Interview Schedule**

This qualitative study will involve individual interviews. Please ensure all participants have read the Patient Information Sheet (PIS) and signed a consent form before their interview.

1. **Interview Schedule: Experience of the PreFIT study and effects of physical activity/exercise on general health and quality of life.**

***Reminder – Purpose of Individual Interviews***

1. To explore participants’ experiences of being involved in the PreFIT study.
2. To explore what physical activity/exercise the participants are currently undertaking and its effects.
3. To identify whether participants physical activity/exercise has changed since participation in PreFIT, and if so, how, and what effect has it had?
4. To explore participants’ views on the barriers and facilitators to physical activity/exercise.

**Before switching on the audio-recorder**

*Background information – why this interview*

“This interview is to help us understand your experience of taking part in the PreFIT study.”

*Confidentiality*

“Before we start talking, I would like to assure you that everything that you say will remain confidential. Your doctor, nurse or any other health professional will not see or hear any of the information that is shared here. If we do use anything that you have said, such as in a report or journal article, it will be made anonymous so that you cannot be identified.”

*Any questions at this stage?*

“Do you have any questions before we switch on the recorder and start?”

**Switch on the audio-recorder**

Interviewees will be encouraged to speak openly and freely about what their experience of the exercise intervention was and how the intervention has affected them.

Indicative questions and prompts are given below (these questions are flexible and not all questions will be appropriate or asked of all participants):

| **Indicative Questions** | **Prompt** |
| --- | --- |
| Can you tell me what made you take part in the PreFIT study?  What were your thoughts and feelings when you were asked?  What was it like for you? | Had you had any falls prior to taking part?  Have you had any since? |
| Have you ever had any falls or problems with your balance?  Can you tell me about what happened when you fell? | To what extent do falls interfere with your life?  Have you ever had any treatment (other than the PreFIT intervention) to help with your balance or falls? |
| Did you have to do any exercises as part of the study?  If yes, what did you think of the exercises?  How did they make you feel? | How did you find doing them?   1. Was there anything easy or hard about doing them? 2. How did you manage with finding the time to do them? 3. Did you like doing them? |
| If no, would you have been interested in doing exercises? Why? | Did you receive the Age UK Staying Steady leaflet?  How did you find the exercise/physical activity information in the leaflet? Was it any help to you? |
| Can you tell me what does exercise, or physical activity mean to you? | What do you think of/picture when someone talks about exercise or physical activity? |
| Can you tell me what effect you think exercise/physical activity has on health? | How does it affect/how has it affected your risk of falling? |
| Do you currently do *any* exercise/physical activity?  If yes, can you tell me about the type of exercise you do.  Why do you do this exercise?  If no, can you tell me why you don’t do any exercise? | Are you including gardening, walking etc. or specific types of exercise? |
| Do you still do any of the exercises you were given as part of PreFIT? Which ones do you do? Why? | Have you altered them? If so, how, and why? |
| Can you tell me about anything that helped you stick with the PreFIT exercises? | What made you want to keep exercising? |
| Can you tell me about anything that made it difficult for you to carry on with or start the PreFIT exercises? | How do these difficulties affect your health/life? |
| What, if anything, prevents you from doing more exercise/physical activity? | What would make it easier for you to be more active? |
| Has the amount or type of exercise/physical activity you do changed since you got involved in the PreFIT study? Why? | What effect has this had on your health/life? |
| What messages would you give to other people who fall or have problems with their balance? |  |
| What messages would you give to other people who are thinking about taking part in a research project? |  |

*Rounding off*

*[Suggested dialogue]*

“Is there something else that you think is important that I haven’t asked you or you would like to discuss? Is there something that you think we should have talked about but haven’t?”

*Thank you*

Thank individuals for their help with the study. We very much appreciate their time.
